# An overview of current research on exercise interventions in aging and aging-related disease

**DOI:** 10.3389/fragi.2026.1832962

**Published:** 2026-05-26

**Authors:** Arad Jain, Campbell Johnston, Yiyu Zhang, Janice Oh, Carolyn Tsung, Eric Kent, Hongji Zhang

**Affiliations:** Department of Surgery, School of Medicine, University of Virginia, Charlottesville, VA, United States

**Keywords:** aerobic training, aging, cognitive function, endurance training, exercise, exercise intervention, functional performance, immune aging

## Abstract

Global declines in physical activity have contributed to an acceleration in immune aging, characterized by systemic inflammation (inflammaging) and impaired immune regulation (immunosenescence). This narrative review provides an overview of the evidence in both preclinical and clinical models supporting exercise as a critical intervention to counteract immune aging and its related diseases. Regular physical activity modulates systemic inflammation, reduces neutrophil extracellular trap (NET) formation, and promotes favorable shifts in immune cell populations, including T cell and natural killer (NK) cell subsets. Exercise interventions have been associated not only with maintaining immune health but also in mitigating autoimmune disease progression, improving metabolic regulation, enhancing tumor immune surveillance, and reducing neuroinflammation. Emerging studies highlight the role of exercise in promoting vascular normalization within the tumor microenvironment, alleviating tumor hypoxia and acidosis, and restoring T and NK cell function. In the elderly, appropriately prescribed multimodal exercise regimens may lower infection risk without clear evidence of immunodepression, supporting exercise as a potentially safe and effective strategy for immune rejuvenation. Furthermore, novel mechanistic insights, including the modulation of NET burden, IGF-1 signaling, kynurenine metabolism, and microbiome composition, suggest that exercise influences key biological pathways underlying age-related immune decline. While exercise offers broad clinical benefits, future research should prioritize mechanistic studies to optimize exercise prescriptions and inform the development of exercise-mimetic therapeutics. Taken together, investigating the exercise regimens employed in these studies remains a promising intervention for promoting healthy immune aging and improving resilience against chronic inflammatory, metabolic, infectious, and malignant diseases.

## Introduction

In the 21st century, changes to work and home environments across the globe have drastically reduced daily physical activity and promoted sedentary leisure activities and occupational tasks (Ng and Popkin, 2012). The World Health Organization (WHO) in 2020 reaffirmed the importance of physical activity, and in response to shifting activity patterns, provided additional recommendations on reducing sedentary behavior. This included specific recommendations for specific populations, including older adults living with chronic conditions or disabilities (Bull et al., 2020). This has become increasingly important in the aftermath of the COVID-19 pandemic (Ráthonyi et al., 2021), as the shift towards working from home has had a significant impact on the increase of sedentary behavior in the working population with a large number of adverse physical and mental health consequences (Morton et al., 2024). One major area of research that is implicated in the decline in physical activity is the relationship between exercise and the immune system. A review in 2019 by Jones and Davison upheld the generally accepted theory that exercise is beneficial for the immune system, with several studies providing clinical and *in vivo* evidence that regular moderate physical activity reduces the risk of serious infection in a wide range of adult populations (Jones and Davison, 2019). This topic is only more relevant now as our workforce ages and life expectancy increases, increasing the cumulative burden of inactivity in our older population. This review is aimed towards investigating the mechanisms by which the immune benefits occur in response to a range of exercise programs within our changing workforce and population. In this review, we also aim to compile data from clinical trials and basic science investigations to evaluate the potential for using exercise therapy within a treatment plan for inflammatory and autoimmune diseases in older adults.

The importance of the long-term effect of this change is increasingly important as the working population ages. Over recent years, life expectancy across the globe has steadily increased (World Health Organization, 2019), and in the US has led to an increase in the average age of retirement and government retirement benefits. The percent of all employed men in the United States that are over the age of 60 rose from 7.4 to 14.8 percent between 2000 and 2020, while similarly it rose from 6.3 to 14.0 percent in women. Primary care physicians are more frequently recommending or prescribing exercise regimens to their patients, and providing guidance for activity, nutrition, and risk reducing strategies (Kettle et al., 2022). This phenomenon has increased particularly with older patients, for which injury risk and physical limitations require greater consideration. However, despite limitations, it’s important to recognize that even low intensity exercise programs below the recommended levels still have benefits in reducing overall mortality risk for elderly patients (Hupin et al., 2015). One potential explanation under investigation has been in suppressing inflammaging, a term coined by Franceschi et al., in 2000 referring to the increased pro-inflammatory burden associated with aging (Franceschi et al., 2000). While it is now well established that this occurs in the setting of generalized immunodeficiency associated with aging, known as immunosenescence (Franceschi et al., 2000; Fagnoni et al., 2000), recent studies have focused on exercise as a modulator of both the inflammatory profiles and functional immunity of an aging population. Recent research has been done to investigate whether exercise can reduce the biochemical metrics associated with a flow cytometry based immune risk profile (IRP) metric which originated from the OCTO/NONA study conducted in Sweden in individuals over 85 years of age (Wikby et al., 2008; Wikby et al., 2009; Pawelec et al., 2010). Recent studies on the benefits of exercise on immune aging are also validated through serum analyses of soluble biomarkers (Aprilia et al., 2024).

In this narrative review, we include studies evaluating the clinical and mechanistic relationships between exercise, immune aging, and aging-related disease (Supplementary Table S1). The review approach was informed by the Joanna Briggs Institute (JBI) checklist/guidance for narrative and textual evidence, with attention to clearly defining the review focus, search strategy, study-selection rationale, and narrative synthesis approach (McArthur et al., 2025). We conducted a literature search of peer-reviewed studies in English or with translation in the PubMed/MEDLINE database through 2026. From that list, we then reviewed studies which focused on older populations. Key search terms include: *“aging,” “older adults,” “exercise,” “physical activity,” “exercise intervention,” “resistance training,” “aerobic training,” “endurance training, “functional performance,” “cognitive function,”* “immune aging,” “immunosenescence,” and “inflammaging,” as well as terms related to the aging-related diseases discussed in this review. Clinical studies were prioritized when they evaluated exercise-based interventions in older adults or in populations with aging-associated conditions and reported clinically relevant, functional, inflammatory, or immune outcomes. A subset of studies that did not exclusively focus on older adults were included when the study addressed risk factors commonly associated with aging, accelerated immune aging, or disease mechanisms relevant to aging biology. Preclinical animal studies and mechanistic studies were incorporated when they provided biological insight into exercise-regulated immune pathways, tissue inflammation, tumor immunity, metabolic regulation, neuroinflammation, neutrophil extracellular trap formation, or microbiome-related effects. Because this article was designed as a narrative review rather than a systematic review or meta-analysis, studies were synthesized narratively to highlight current evidence, identify mechanistic themes, and define promising directions for future translational and clinical investigation.

Although the review is organized by disease context for clarity, aging biology and immune aging remain the central framework through which these conditions are discussed. Each section is therefore intended to highlight how exercise may influence shared mechanisms of immunosenescence, inflammaging, and age-related immune dysfunction across different clinical settings.

## Exercise and systemic inflammation

A study published in 2021 by Despeghel et al. investigated the effects of a 6-week, American College of Sports Medicine guideline compliant, combined resistance and endurance exercise program on the IRP scores for previously sedentary healthy adults over the age of 60 (Despeghel et al., 2021). The intervention level was sufficiently accessible such that all participants were able to complete all training sessions. Flow cytometry analysis of participants in the training arm of the study showed an increase in the CD4+/CD8+ ratio, indicating an improvement in IRP (Wikby et al., 2009; Despeghel et al., 2021). However, this program did not lead to significant changes in CD4^+^ or CD8^+^ subpopulations that are typically associated with aging in the IRP. Instead, they conducted serum studies and found a reduction in the interleukin (IL)-6 mediated proinflammatory state, with no changes in tumor necrosis factor alpha (TNF-ɑ) mediated inflammaging as these participants had no baseline inflammatory disease (Despeghel et al., 2021; Bautmans et al., 2021).

Previous studies had separated resistance and aerobic training groups and enrolled participants in longer training programs. One such study previously healthy women over the age of 60 conducted 6 months training programs, separating aerobic from resistance groups and similarly found a reduction in the IL-6 and TNF-ɑ mediated proinflammatory state, with an increase in anti-inflammatory IL-10 (Abd El-Kader and Al-Shreef, 2018). However, while they did see an increase in both CD4^+^ and CD8^+^ cell counts, they did not appreciate any increase in the CD4+/CD8+ ratio. Nevertheless, these exercise benefits are still appreciable in the absence of any weight change, as has been shown in previous studies as well (Nicklas et al., 2008). Recent studies of Functional Training (FT) and Combined Training (CT) protocols in a cohort of post-menopausal women demonstrated notable reductions in the proportion of CD4^+^ Terminally Differentiated Effector Memory T Cells Re-Expressing CD45RA (TEMRA) and CD8^+^ TEMRA cells. Moreover, both FT and CT contributed to increased percentages of central memory (TCM) CD4^+^ and CD8^+^ T cells, as well as enhanced levels of CD8^+^ effector memory T cells (TEM). While these training approaches were shown to lead to improvements in various measures of physical performance, the findings suggest that both FT and CT are equally viable strategies for enhancing immune cell profile dynamics and functional fitness in this demographic. Such interventions hold promise in addressing immunosenescence in aging populations (Vasconcelos et al., 2022).

While cell population studies are useful in showing the importance of exercise in immune system maintenance, serum biomarkers may possess greater clinical utility (Aprilia et al., 2024). Additional general inflammatory mediators have shown a systemic response to exercise. A study in 2021 by Hayes et al. compared lifetime exercisers with previously sedentary adults as both groups underwent a preconditioning exercise program over 6 weeks, followed by 6 weeks of high intensity interval training (HIIT). As in previously mentioned studies, preconditioning was able to diminish the IL-6 proinflammatory state as well as decrease systemic inflammation through a decrease in c-reactive protein (CRP), with no change in homocysteine mediated pathways (Hayes et al., 2021). Avoiding premature senescence of the immune system was tracked in a separate study of younger adult participants engaging in a 12-week physical activity (Fitbit Tracked) intervention. This intervention mitigated the elevated p16INK4a levels in PBMCs, though it showed no effect on p21Cip1 and senescence-associated secretory phenotypes. Objectively measured moderate–vigorous physical activity was independently and inversely correlated with the expression of p16INK4a and p21Cip1 in the peripheral blood mononuclear cell (PBMCs) of adults with obesity (Chen et al., 2022). Physical inactivity was an independent determinant of premature senescence in immune cells, irrespective of chronological age, body mass index, body fat, maximal oxygen consumption, sedentary behaviors, and sleep duration. This 12-week intervention highlights a promising strategy to mitigate premature immune senescence in adults with obesity earlier in life, which only compounds with typical rates of age-related decline in immune function. Further, this highlights the p16INK4a and p21Cip1 genes as future targets for research in immunosenescence.

In addition, recent studies have implicated the formation of neutrophil extracellular traps, or NETs, in the pathogenesis of inflammaging and aging related immune deficiency such as atherosclerosis, vascular disease, autoimmune/autoinflammatory disease, metabolic disease, malignant neoplasia, sepsis, and critical illness due to COVID-19. Neutrophil extracellular traps function to capture and slow down the spread of pathogens through release of decondensed DNA with bactericidal proteins (Nathan, 2006; Papayannopoulos, 2018; Nirmala and Lopus, 2020; Pérez-Figueroa et al., 2021). *In-vitro* and animal models investigating the interaction of neutrophils with activated platelets or endothelial cells correlate with several immunologic and systemic triggers involved in these disease processes, including hyperglycemia, nitric oxide, urate crystals, autoantibodies, proinflammatory cytokines such as IL-1β, IL-6, IL-8, TNF-α (Klopf et al., 2021). This process has significant interplay with the phenomena described above as presence of NETs in plasma predicts the occurrence of multiple organ failure, sepsis, and tissue damage in SIRS patients. Studies demonstrate the ability of TNFα, IL-1β, and IL-8 in eliciting neutrophils oxidative burst and NETs formation. Elevated levels of these cytokines in the circulation, as well as NET formation, seem to be involved in the pathology of SIRS (Keshari et al., 2012; Custodero et al., 2020), a relatively common risk associated with older individuals with infectious agent exposure and those undergoing procedures (Chakraborty and Burns, 2024; Gokalp et al., 2018). Novel research has clearly demonstrated NETs as a significant avenue by which exercise can prevent and reverse the formation of proinflammatory phenotypes associated with aging. In a study of both younger and older men undergoing a 12 weeks HIIT regimen, it was reaffirmed that the serum of older men induces a higher rate of baseline NET proliferation as compared to younger males. However, a HIIT regimen was able to reduce the rate of NETosis observed through live cell imaging in the older adults, indicating that exercise is an effective method of modulating the development of NETs in an aging population (Vidal-Seguel et al., 2023). In a separate study, ninety-eight adults aged 30 to 65 with cardiovascular disease risk factors participated in an 8-month exercise study. Prior to starting, baseline immune and fitness assessments were conducted, with measurements taken at intervals to avoid immediate post-exercise inflammation spikes. Comparing baseline to the 8 month mark, researchers found reduced circulating cell-free DNA (cfDNA) levels and increased DNase activity, particularly among those improving physical performance. Regular exercise adherence helped lower NET burden and mitigate pro-inflammatory signals, attributed to elevated DNase activity induced by exercise (Ondracek et al., 2022). From these studies, it is clear that exercise can be a key element of a comprehensive treatment plan for disease processes where these cell, cytokine, and neutrophil based immunological phenomena are implicated, but further study is needed to standardize regimens to fully evaluate this paradigm in a clinical setting.

## Exercise and autoimmune disease

These factors are increasingly important when considering the number of inflammatory disease processes associated with aging. Immunosenescence is a major risk factor for autoimmune disease, particularly when T cell generation and immune regulatory checkpoints are more susceptible (Goronzy and Weyand, 2012). A comprehensive review of rheumatological and musculoskeletal diseases showed that exercise interventions resulted in improvements in outcomes such as pain and function across all the diseases studied, although the size of the effect varied by autoimmune disease and intervention. Disease activity was not influenced by exercise, other than in axial spondyloarthritis. Increased body weight was associated with worse outcomes for the majority of diseases and outcomes assessed. In general, study quality was moderate for the literature on exercise and body weight in autoimmune disease, although there was large heterogeneity between studies. Patients included those with osteoarthritis, rheumatoid arthritis, systemic lupus erythematosus, axial spondyloarthritis, psoriatic arthritis, systemic sclerosis and gout (Gwinnutt et al., 2022). As stated before, many of these processes are T-cell mediated and susceptible to T-cell senescence and dysregulation. A 6-week low intensity strength endurance exercise regimen, in a study of 100 women over the age of 65, significantly decreased the basal percentage and absolute counts of senescence-prone T cells, which was positively correlated with the number of training sessions performed (Cao Dinh et al., 2019). Other idiopathic immune diseases, such as irritable bowel diseases (IBD) like Ulcerative Colitis and Crohn’s, generally arise in a younger population, but have a bi-modal distribution, with a second peak incidence occurring between the age of 60–80. However, macrophage dysregulation and telomere dysfunction have been intrinsically linked to decreased gut wall integrity and the development of IBD (Sienkiewicz et al., 2023). This is indicative of a premature immune aging state associated with these autoinflammatory diseases. In fact, this year, in a mouse model of Ulcerative Colitis, 1-h daily treadmill exercise independently and in conjunction with 5-ASA enema treatments suppressed pro-inflammatory cytokines and apoptosis. The authors showed that benefits of this simultaneous treatment may be due to inhibition on nuclear factor-κB/mitogen-activated protein kinase signaling activation, leading to TNFα toxicity mediated apoptosis (Jin et al., 2024).

Another potential route by which an exercise regimen can reduce the severity and progression of autoimmune and inflammatory diseases is through reducing the rate of uncontrolled NETosis, or NET formation. This pathology has been a focus of recent study in the field of exercise and immunology research. NETs are a significant factor in the pathogenesis of idiopathic autoimmune disorders (Liu R. et al., 2024), and have been shown to further stimulate the inflammatory response of RA (Khandpur et al., 2013). Medical therapies that target NET formation such as peptidylarginine deiminase and triptolide have already shown efficacy in mouse models of rheumatoid arthritis, systemic lupus erythematosus, and vascular disease (Kni et al., 2013; Knight et al., 2014; Liu P. et al., 2024). Exercise is able to reverse neutrophil mediated pathology in systemic inflammaging (Vidal-Seguel et al., 2023; Ondracek et al., 2022), a phenomenon shared with these disease processes. In a study of 26 irritable bowel patients vs. controls, a 12-week walking activity program improved conditioning and raised the counteracting activity of DNase against pro-inflammatory signaling. Further, this study indicated a potential imbalance in cell-free mitochondrial DNA clearance, highlighting a potential avenue for future study (Chimienti et al., 2024). As exogenous DNase is being investigated as a therapy for a multitude of inflammatory diseases in both human studies of inflammatory disease and mouse models of steatohepatitis (Lauková et al., 2020; Wu et al., 2023), using exercise as a method of activation of endogenous DNase can potentially be effective in managing chronic inflammatory illness. Exercise has also been shown to induce microbiome changes that can contribute to a reduction in IBD severity. A study of previously sedentary adults showed that sprint interval and moderate continuous exercise programs can increase Bacteroidetes and decrease Firmicutes/Bacteroidetes ratio (Motiani et al., 2020). This study corroborated previous studies in murine models (Evans et al., 2014; Lambert et al., 2015). As relative abundance of Bacteroidetes is inversely correlated with disease activity in IBD, exercise could potentially serve as an effective component of disease management (Nomura et al., 2021). Further studies are needed on whether this same paradigm applies to the subset of patients with IBD that are diagnosed within the second peak of the bimodal incidence distribution. Whether exercise interventions can help mitigate symptoms or even prevent the development of these inflammatory syndromes in the 60–80 year old age group would be worthy of future study.

## Exercise and metabolic regulation of the immune system

Metabolic syndrome is an inherently inflammatory cluster of risk factors for cardiovascular disease associated with obesity and type II diabetes. The earliest studies of metabolic syndrome identified weight, age, lifestyle and caloric intake as the major non-genetic determining factors in development of metabolic syndrome. Early aerobic exercise studies dating back to 2003 showed that a 20 weeks exercise regimen was able to improve measurable outcomes such that 30% of participants were no longer classified as having metabolic syndrome after the intervention (Katzmarz et al., 2003). In the following years, it became more accepted that age is not only a risk factor for metabolic syndrome, but that metabolic syndrome also induces precocious aging (Bonomini et al., 2015). Mammalian target of rapamycin (mTOR), AMP-activated protein kinase (AMPK), sirtuins and insulin/insulin like growth factor 1 (IGF-1) signaling have been implicated in both cell protection and lifespan and in disruptions to substrate and energy homeostasis associated with metabolic syndrome, a process both accelerated by and contributing to premature aging (Kenyon, 2010). In particular, numerous studies have shown that chronic inflammation suppresses the growth hormone/IGF-1 pathway through increased levels IL-6, TNFα, and IL-1 β (Witkowska-Sędek and Pyrżak, 2020), accelerating the age-related decline in IGF-1 and increasing the risk of cognitive decline and death (Arai et al., 2001; Cappola et al., 2003). A study of younger healthy adults in 2020 showed that there is an immediate but short lived spike in free IGF-1 after exercise, with a return to baseline within 24 h (Gulick et al., 2020). However, in a cohort of 60 elderly adults with sarcopenic obesity, an 8 weeks resistance or resistance/aerobic combination training program was able to increase physical performance and resting IGF-1 serum levels, and suggest that some potential benefits may still persist 4 weeks after the training program (Chen et al., 2017). This again highlights the importance of resistance and endurance exercise, particularly in an aging population with metabolic risk factors and disease. Diabetes directly impacts skeletal muscle thus contributing to mobility reduction in older individuals and reduces functional capacity through impaired muscle function (Angulo et al., 2020). Further, this association has been shown to correlate with insulin resistance (Barzilay et al., 2007; Yang et al., 2017). It is clear that there is a significant correlation between metabolic syndrome and frailty, particularly in the age range of 50–65 (Angulo et al., 2020), suggesting that metabolic syndrome appears to accelerate the biological aging process through systemic inflammatory processes and hormonal balance changes.

Even prior to the development of metabolic syndrome, obesity leads to metabolic and inflammatory changes that are reversible by exercise. Early studies showed that 12 weeks of combination exercise training, more so than aerobic or resistance exercise, decreased TNF-α in overweight and obese individuals compared to no exercise, indicating that combination exercise training may be physiologically relevant in decreasing the risk of diabetes and cardiovascular disease (Ho et al., 2013). Additionally, while it is well known that exercise can reduce weight gain through increasing calorie consumption, subsequent studies suggested that exercise can not only prevent weight gain, but also increase Bacteroidetes populations and decrease the Firmicutes/Bacteroidetes ratio in a mouse model of high fat diet induced obesity after 12 weeks, much like as described with IBD (Chen et al., 2017).

Additional pathways that may explain the immunomodulatory metabolic effects of exercise have been the focus of recent studies. In a study of healthy adult males, single bouts of endurance exercise caused acute changes in the kynurenine pathway, inducing more significant effects than resistance exercise. Endurance exercise enhances the conversion of kynurenine to kynurenic acid and quinolinic acid, suggesting improved peripheral kynurenine clearance and reduced CNS accumulation. The kynurenine/tryptophan ratio positively correlates with IL-6 and CD56^bright^ NK cells, while negatively correlating with CD56^dim^ NK cells. Endurance exercise also increases kynurenic acid levels, quinolinic acid levels, and KAT4 expression post-exercise (Joisten et al., 2020). Additionally, recent studies in mouse models have shown that 4 weeks of exercise can induce HMGB1 release to enhance itaconate metabolism in the TCA cycle. Thereby, exercise metabolically reprograms Kupffer cells into an anti-inflammatory phenotype that can significantly attenuate liver inflammation from ischemia/reperfusion injury via the Nrf2 pathway. Potential mechanisms such as these serve as a starting point for investigating exercise-mimicking pharmaceutical candidates to protect against liver injury during surgery (Zhang et al., 2021). Overall, there are a wide variety of ways in which the enteric immune system responds to exercise, each of which promotes a healthy aging phenotype in adults.

## Exercise and tumor immune microenvironment

Aging of the immune system is inextricably linked with increased risk and poorer outcomes of many cancers, as the peak incidence of cancers in the seventh and eighth decades of life coincides with the peak of immunosenescence (Fulop et al., 2009). This occurs due to a large number of factors, including changes in T cell populations (Foster et al., 2011), natural killer (NK) cell efficacy (Hazeldine and Lord, 2013), and accumulation of reactive oxygen species (ROS) (Kudryavtseva et al., 2016).

NK cells have been shown to have anti-cancer effects and can modulate the immune environment (Crinier et al., 2020). Exercise intervention studies increased survival via improved mobilization and infiltration of NK cells into B16F10 melanoma mouse tumors (Pedersen et al., 2016). Schlagheck and colleagues have also shown exercise to be associated with immune cell mobilization in human studies (Schlagheck et al., 2020). Interleukin 6 (IL-6) is a cytokine thought to be positively correlated with NK cell distribution. Bay and colleagues showed in their 2020 study that inhibition of IL-6 receptors greatly reduced NK cell mobilization into circulation in response to exercise; peak circulating NK cells dropped 53% (Bay et al., 2020). This suggests that IL-6 may be responsible for the increased NK cell counts observed following exercise, as IL-6 has been shown to be increased in plasma following aerobic exercise (Steensberg et al., 2000). Together, these findings support a potential role for exercise-induced NK cell mobilization in antitumor immunity, although direct evidence in older adults and aging-related cancer populations remains limited.

Exercise is further significant for mounting an improved immune response via its impact of NK cells in hypoxic conditions. Inactivity and aging are both independently associated with cellular hypoxia, however, due to the hypoxic nature of the tumor microenvironment (TME), pre-clinical evidence suggests that NK cell function may be further reduced at rest (Balsamo et al., 2013). Following an acute bout of exercise, even in younger but previously inactive adults, NK cell cytotoxicity was restored to near normoxic levels when cocultured with triple-negative breast cancer cells (Cho et al., 2023).

Exercise training also enhances the antitumor effects of CD8^+^ T cells. CD8^+^ T cells are mobilized into tumors through the interactions between CXCR3 and CXCL9, 10 and 11 (House et al., 2020; Tian et al., 2017; Maurice et al., 2019). In mice with human tumor xenografts, interferon-γ (IFN-γ) induces the expression of CXCL9, 10 and 11, driven by CD8^+^ T cells following exercise. This may promote a feed-forward process involving chemokine induction, T cell recruitment, and improved antitumor immune activity. Exercise-catalyzed tumor infiltration of CD8^+^ T cells can also be augmented via exercise-induced increases in epinephrine (EPI) levels in mouse models, and clinical trials have shown promising data of similar phenomena in patients with lung cancer (Holmen et al., 2022; Miao et al., 2024). These findings suggest that exercise-regulated adrenergic and chemokine pathways may support CD8^+^ T cell recruitment, although additional studies are needed to determine whether this mechanism is preserved in aging human populations.

Beyond immune-cell recruitment, preclinical studies suggest that exercise may influence tumor vascularization, hypoxia, and immune-checkpoint responsiveness. Hypoxia and abnormal tumor vasculature can restrict immune-cell infiltration and impair delivery of immunotherapies, while vascular normalization may improve T cell entry and therapeutic response (Dvorak et al., 1988; Yu et al., 2021; Huang et al., 2012; Allen et al., 2017). These mechanisms are highly relevant to aging-associated cancer biology, but they remain largely preclinical and should be interpreted as hypothesis-generating.

In general, hypoxia is a state associated with aging, as well as many aging-related disease states. With aging, the body’s natural ability to break down ROS also declines, which puts patients at further risk of cancer and poor outcomes from cancer treatment (Nisar et al., 2024). Hypoxia further hampers the efficacy of anti-PD-1 therapies (as well as other immunotherapies) via tumoral acidosis. Experimental studies suggest that acidic tumor conditions can impair CD8^+^ T cell function and that restoring physiological pH may improve antitumor immune responses (Calcinotto et al., 2012; Fischer et al., 2007; Pilon-Thomas et al., 2016). This suggests that T cell function can be rescued following restoration of physiological pH, thus increasing immunotherapy efficacy. Exercise has shown promise as a means to reduce intratumoral hypoxia and enhance immunotherapy efficacy. Betof and colleagues demonstrated in preclinical mouse models that indicators of improved vasculature, namely microvessel density, vessel maturity and perfusion, were all increased in exercise vs. sedentary groups. This was correlated with reduced intratumoral hypoxia, suggesting that exercise can result in the generation of stronger vasculature as a means to decrease TME hypoxia (Betof et al., 2015). Therefore, exercise-related vascular and metabolic remodeling may support antitumor immunity, but this remains to be validated in older patients receiving immunotherapy.

Tumoral acidosis is similarly alleviated with exercise in preclinical models. Avesh and colleagues showed in their 2015 study that mice undergoing endurance training exhibited lower LDH-A expression within MC4-L2 (an injectable murine breast carcinoma cell line) breast tumors. This was correlated with decreased lactate concentration in those tumors as well, indicating a decrease in intratumoral acidity (Aveseh et al., 2015). Consequently, these preclinical studies suggest that exercise may reduce hypoxia-associated tumor acidosis and thereby support antitumor immune activity. However, these findings remain primarily preclinical and require validation in aging human cancer populations before being extrapolated to clinical exercise prescriptions or immunotherapy combinations.

## Exercise and neuroinflammation

Dementia is one of the most significant concerns in aging populations, and has been recently reported to be as prevalent as 22%–55% in clinical scenarios, and up to and over 80% of care home residents around the world (Davis et al., 2021; Kao et al., 2022; Fagundes et al., 2021). Alzheimer’s dementia is regarded as one of the most difficult conditions to treat, with exercise often showing little benefit (Lamb et al., 2018a; Lamb et al., 2018b). However, some studies have shown that exercise may help protect patients with predementia through modulation of amyloid β turnover, inflammation, synthesis and release of neurotrophins, and improvements in cerebral blood flow (De La Rosa et al., 2020; Qi et al., 2021). In other neurodegenerative diseases such as Parkinson’s, exercise has more clearly shown clinical benefit and is the focus of many large clinical trials and community programs (Alberts and Rosenfeldt et al., 2020; Moore et al., 2021). In contrast to the progressive diseases, aging and age related diseases are major risk factors for stroke. Exercise and rehabilitation are widely accepted as key components of stroke recovery, as well as reducing risk (Lee et al., 2022). With stroke recovery in particular, constraint induced movement therapy has shown to reduce NET formation in mouse models similar to pharmacologic intervention (Li et al., 2023). This study showed that physical rehabilitation was successful in reducing the effects of this neutrophil driven mechanism of blood brain barrier dysfunction. These findings suggest that exercise or rehabilitation may influence neuroinflammation through mechanisms that overlap with systemic inflammatory regulation, although additional studies are needed to define how these pathways operate in aging human populations.

Clinical and cognitive outcomes have been evaluated in several exercise-based studies involving older adults. One study evaluated 6 months of chair-based strength and multimodal exercise in frail and pre-frail care home residents with cognitive impairment as measured by Mini-Mental State Examination (MMSE). The multimodal exercise intervention showed improvements in physical performance and cognitive measures, with changes in immune parameters correlating with improvements in physical and cognitive performance (Eustáquio et al., 2020). Other studies of the anti-inflammatory effect of a 14-week exercise and taurine supplementation have shown improvements in cognitive outcomes in elderly women, with MMSE score increasing only in the exercise plus taurine group (Chupel et al., 2018).

In the chair-based strength and multimodal exercise study, the multimodal intervention was associated with a moderate decrease in the TNF-α to IL-10 ratio, significant time-by-group interactions with salivary IgA and IL-10, and slight reductions in IL-6 and IL-1β concentrations (Eustáquio et al., 2020). In the exercise and taurine supplementation study, the exercise regimen reduced TNF-α, IL-6, and IL-1β/IL-1ra, IL-6/IL-10, and TNF-α/IL-10 ratios (Chupel et al., 2018). These findings suggest that exercise may attenuate pro-inflammatory signaling and improve inflammatory balance in older adults, although the specific contribution of exercise alone may vary depending on study design and combined interventions.

Blood-brain barrier-related biomarkers provide another potential link between exercise, aging, and neuroinflammation. Serum concentration of S100β, a protein marker associated with intracranial lesions and decreased blood brain barrier integrity (Egea-Guerrero et al., 2012; Koh and Lee, 2014), was maintained with intervention but increased in non-exercise, non-supplemented controls, while neuron specific enolase levels were only raised with taurine but not exercise (Chupel et al., 2018). S100β has been implicated in several systemic proinflammatory processes, and may provide a potential mechanism to explain some of the benefits that exercise may have in this patient population (Xia et al., 2018; Michetti et al., 2023). Together, these studies suggest that exercise-based interventions, even when initiated in very old adults, may influence cognitive function, inflammatory signaling, and markers related to blood-brain barrier integrity. However, further studies separating cognitive endpoints, inflammatory biomarkers, and blood-brain barrier-related biomarkers will be important for defining the specific mechanisms by which exercise may support neurological health in aging populations.

## Exercise and infection risk in the elderly

Exercise has clearly demonstrated a significant positive effect on systemic and local inflammation in the aging population. However, due to weakened immune systems and increased risk of spreading infection through care homes, there is some controversy regarding the risks of infection during exercise. Studies of athletes and other individuals who train at high intensity or long durations have shown an apparent risk of contracting upper respiratory infections. This is likely related to regular acute periods of exercise-induced immunodepression (Jones and Davison, 2019). If this phenomenon is present in a population that has physiologically adapted to regular exercise, it is important to make sure that it is mitigated in the older adult population when starting exercise based therapies.

Studies have shown that at submaximal loads with older patients, as 50% and 80% of maximal resistance, when performed after an adaptation period, did not induce injuries and were well tolerated by the participants without negative acute effects on blood cortisol, total leukocytes, lymphocytes and their subpopulations, and salivary IgA (Neves et al., 2009). In an 8-month clinical trial involving participants over 60, moderate exercise did not impact the risk or severity of respiratory infections (RIs) compared to a control group maintaining daily physical activities. Metabolomic profiling revealed differences in serum metabolites, such as alcohols, ketones, alkanes and aldehydes, between the exercise and control groups. Despite these metabolic changes, the exercise regimen was not associated with increased susceptibility to RIs, suggesting that transient immunosuppression due to exercise is not a significant concern in older adults participating in moderate intensity 90-min training sessions three times/week. However, since a pattern of lipid peroxidation was associated with the number of RIs, there are tools to monitor individuals who are still believed to be at an elevated risk (Silva et al., 2019).

Overall, while these concerns have shown validity in certain populations, with careful monitoring of immune health and maintaining a moderate submaximal load during the training program, it is very possible to minimize the risk of infection while using exercise-based therapies for the elderly population. However, to ensure the safety of our older population, safety thresholds for moderate exercise may still differ with respect to exercise intensity, frailty, nutrition status, comorbidity, and institutional living environment, independent of the lack of increased infection risk.

Exercise interventions modulate immune status by suppressing proinflammatory signaling TNF-α, IL-6, IL-1β, CRP and cellular senescence markers p16INK4a. These interventions also promote anti-inflammatory responses, including increased IL-10 and salivary IgA. Attenuation of NETosis is observed, accompanied by increased DNase activity and reduced circulating cell-free DNA levels. Exercise further induces systemic adaptations, including increased IGF-1 levels and modulation of gut microbiota composition, reflected by a higher Bacteroidetes/Firmicutes ratio, consistent with a metabolically healthy state. In parallel, exercise enhances CD8^+^ T cell and NK cell infiltration, mediated in part by elevated epinephrine and activation of the kynurenine pathway, reduces senescence-prone T cell populations, and improves T cell memory profiles, characterized by decreased terminally differentiated TEMRA cells and increased central and effector memory T cells. Exercise also reduces oxidative stress through thioredoxin-mediated scavenging of H_2_O_2_ and alleviates tumor hypoxia.

Exercise in preclinical models suppresses proinflammatory TNF-α signaling and attenuates NETosis through increased DNase activity. Exercise also induces an anti-inflammatory phenotype in Kupffer cells through HMGB1-mediated enhancement of itaconate metabolism. In parallel, exercise enhances antitumor immunity by promoting CD8^+^ T cell infiltration into tumors via CXCR3-CXCL9/10/11 signaling, with CD8^+^ T cell-derived IFN-γ further amplifying chemokine expression to establish a positive feedback loop. Exercise further modulates gut microbiota composition, including increased Bacteroidetes abundance and Bacteroidetes to Firmicutes ratio in obesity models. Additionally, exercise reduces oxidative stress through thioredoxin-mediated scavenging of H_2_O_2_, alleviates tumor hypoxia, and may promote vascular normalization, potentially improving drug delivery. Exercise also decreases LDH-A expression and intratumoral lactate accumulation, leading to reduced tumor acidosis.

## Limitations

Several limitations of the current field should be considered when interpreting the literature summarized in this review. First, substantial heterogeneity exists across studies with respect to exercise modality, intensity, frequency, duration, and adherence, making direct comparison difficult and limiting the ability to define optimal regimens. Second, the populations studied are highly diverse, ranging from healthy older adults to patients with frailty, obesity, autoimmune disease, cancer, and neurodegenerative conditions, each with distinct biological and clinical contexts. Third, many intervention studies are limited by relatively small sample sizes, short follow-up periods, and a lack of standardized immune endpoints. Finally, although mechanistic studies have generated important insights, many of these findings remain incompletely translated into clinical settings. Together, these limitations highlight the need for more standardized, longitudinal, and mechanistically integrated clinical studies.

## Conclusion

**FIGURE 1:**
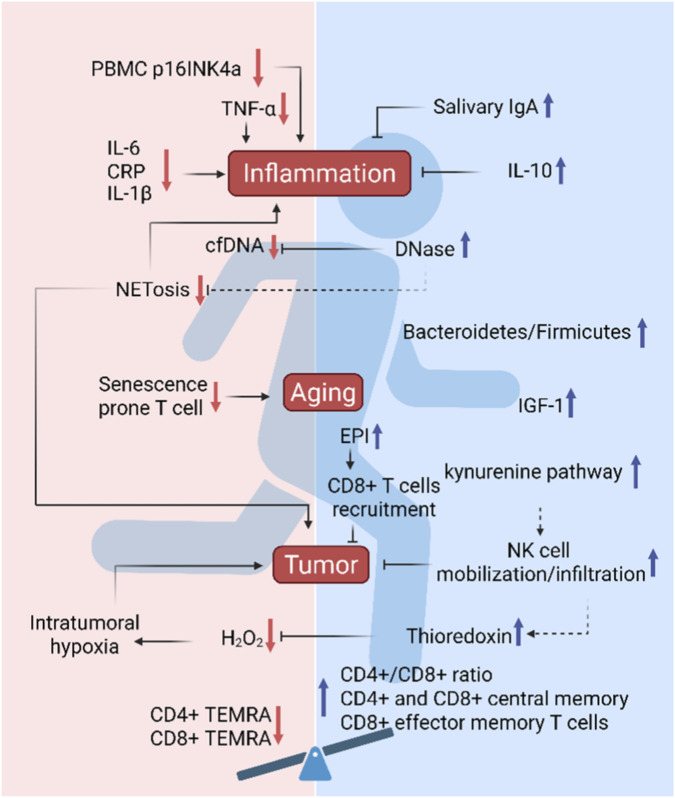
Molecular pathways involved in inflammation, aging, and tumor-related immune responses modulated by exercise in humans. PBMC, peripheral blood mononuclear cell; TNF-α, tumor necrosis factor-alpha; IL-6, interleukin-6; CRP, c-reactive protein; cfDNA, cell-free DNA; IGF-1, insulin-like growth factor 1; EPI, epinephrine; NK cell, natural killer cell; TEMRA, terminally differentiated effector memory T cells.

**FIGURE 2:**
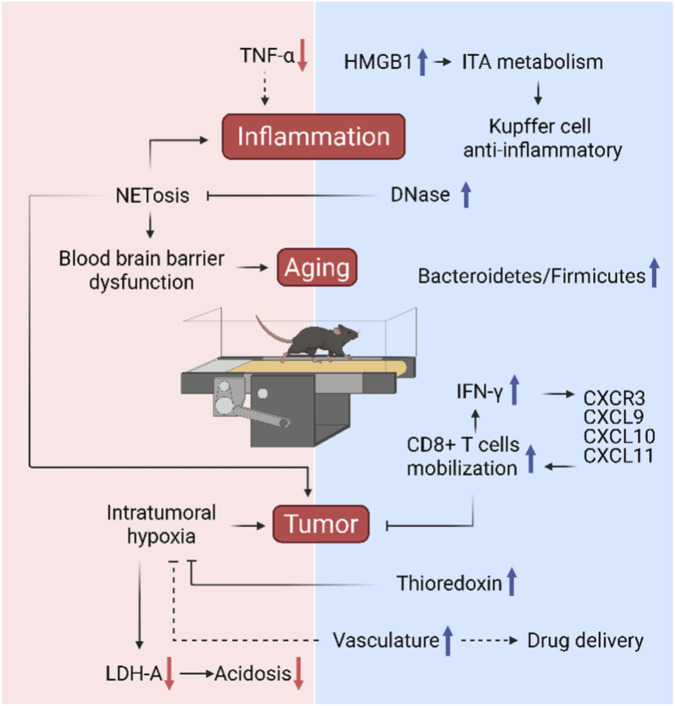
Molecular pathways involved in inflammation, aging, and tumor-related immune responses modulated by exercise in preclinical studies. TNF-α, tumor necrosis factor- alpha; HMGB1, High-mobility group box 1 ; ITA, itaconate; IFN-γ, Interferon gamma; CXCR3, CXC motif chemokine receptor 3; CXCL9, C-X-C motif chemokine ligand 9; LDH-A, lactate dehydrogenase A.

Overall, evidence in clinical and animal studies has shown a potential role of exercise in improving systemic inflammation and immunosuppression associated with aging, as well as several age-related diseases in both animal and human studies (Figures 1, 2). In each of these categories, it has been shown that low-moderate intensity, multimodal combined resistance and aerobic exercise can be effective within these study populations while reducing any unnecessary risks when performed in a controlled setting.

One future direction of exercise research needs to be refining and optimizing the training programs for this patient population to maximize efficacy, safety, and motivation. Our ability to draw conclusions from the current body of research is significantly limited by a lack of standardization in exercise modality, intensity, frequency and duration. It is consequently difficult to ascertain a causal relationship between the interventions and outcomes without a pattern across a broad data set that elucidates a dose-response interaction between exercise and immune aging phenomena. Particularly with aging research, long term follow-up of participants, particularly in the studies with younger cohorts, is important to investigate. However, this is a significant limitation of the studies reviewed and necessitates further study. The applicability of these studies is also limited in patients who are exercise intolerant, though there are groups that have recently been bridging this gap in their research. Studies of frail and institutionalized elderly adults (aged 84.8 ± 7.9) showed improvement in cognitive state via MMSE and Pfeiffer and Barthel tests, and functional independence, while in the physical and mental component of the S-12, significant improvement was generated (Mollinedo et al., 2019). Further, efforts have been made in developing training programs for elderly patients with sarcopenia, demonstrating kettlebell training significantly increases the sarcopenia index and strength, with the retention effect of the training program continuing after 4 weeks of detraining (Chen et al., 2018). While standardization would be helpful from a research perspective, work that continues to evaluate a range of training modalities for this patient population will be essential to the health of our community in the future.

However, even in the treatment of patients who are unable to exercise, research that uses exercise as an intervention has and can continue to elucidate mechanisms that can be targets of other modes of treatment. There is a significant gap between these mechanistic studies and clinical applications, particularly in cases where these targets have a wide range of roles in the human body, many of which are beneficial. Further studies investigating the mechanism by which exercise can suppress TNF-ɑ and IL-6 mediated systemic inflammation without leaving the patient in an immunocompromised state will be essential to future treatments for inflammaging. Translational research efforts on these mechanistic targets have fortunately gained popularity. For example, neutrophil extracellular trap formation (NETosis), has already been the target of pharmacological intervention through use of deoxyribonucleases (Lauková et al., 2020), and microbiome focused therapeutics are also showing promising developments (Gulliver et al., 2022). Further still, studies of additional pathways mediated by IGF-1, S100β, Kynurenine, HMGB1, p16INK4a and p21Cip1 mentioned previously in this review may bring us closer to replicating and enhancing the effectiveness of exercise based therapy in both healthy aging populations and those with chronic disease. For now, however, it is important to continue to emphasize exercise into treatment programs to any extent possible due to the wide range of systemic and local benefits with limited significant risk.
